# Diabetic status and the relation of the three domains of glycemic control to mortality in critically ill patients: an international multicenter cohort study

**DOI:** 10.1186/cc12547

**Published:** 2013-03-01

**Authors:** James S Krinsley, Moritoki Egi, Alex Kiss, Amin N Devendra, Philipp Schuetz, Paula M Maurer, Marcus J Schultz, Roosmarijn TM van Hooijdonk, Morita Kiyoshi, Iain MJ Mackenzie, Djillali Annane, Peter Stow, Stanley A Nasraway, Sharon Holewinski, Ulrike Holzinger, Jean-Charles Preiser, Jean-Louis Vincent, Rinaldo Bellomo

**Affiliations:** 1 Division of Critical Care, Stamford Hospital and Columbia University College of Physicians and Surgeons, 190 West Broad Street, Stamford, CT, 06902, USA; 2 Department of Anesthesiology and Resuscitology, Okayama University Hospital, 2-5-1 Shikatachou, Okayama, 700-8525, Japan; 3 Institute of Health Policy, Management and Evaluation, University of Toronto, 155 College Street, Toronto, M5T 3M6, Ontario, Canada; 4 Medical/Surgical Intensive Care Unit, Morton Plant Hospital, 300 Pinellas Street, Clearwater, FL 33756, USA; 5 Privat Dozent for Endocrinology and Internal Medicine, Medical University Department, Kantonsspital Aarau, Tellstrasse CH -5001 Aarau, Switzerland; 6 BayCare Health Systems, 300 Pinellas Street, Clearwater, FL 33756, USA; 7 Department of Intensive Care, Academic Medical Center, Meibergrdeef 9, 1105AZ, Amsterdam, The Netherlands; 8 Department of Anesthesia and Critical Care Medicine, University Hospital Birmingham NHS, Mindelsohn Way, Edgbaston, B15 2WB, Birmingham, UK; 9 Critical Care Department, Service de Réanimation, Hopital Raymond Poincaré, Université de Versailles SQY, 104 Boulevard Raymond Poincare, 92830, Garches, France; 10 Intensive Care Unit, The Geelong Hospital, Barwon Health, Ryrie Street, Geelong, Victoria, 3220, Australia; 11 Surgical Intensive Care Units, Tufts Medical Center, 800 Washington Street, NEMC 4360, Boston, MA 02111, USA; 12 Medical Intensive Care Unit, Department of Medicine III, Division of Gastroenterology and Hepatology, ICU 13H1, Medical University of Vienna, Waehringer Guertel 18-20, Vienna, 1090, Austria; 13 Department of Intensive Care, Erasme University Hospital, Université Libre de Bruxelles, Route de Lennik 808, Brussels, 1070, Belgium; 14 Department of Intensive Care, Austin Hospital and Monash University, Studley Road, Heidelberg, Victoria, 3084, Australia

## Abstract

**Introduction:**

Hyperglycemia, hypoglycemia, and increased glycemic variability have each been independently associated with increased risk of mortality in critically ill patients. The role of diabetic status on modulating the relation of these three domains of glycemic control with mortality remains uncertain. The purpose of this investigation was to determine how diabetic status affects the relation of hyperglycemia, hypoglycemia, and increased glycemic variability with the risk of mortality in critically ill patients.

**Methods:**

This is a retrospective analysis of prospectively collected data involving 44,964 patients admitted to 23 intensive care units (ICUs) from nine countries, between February 2001 and May 2012. We analyzed mean blood glucose concentration (BG), coefficient of variation (CV), and minimal BG and created multivariable models to analyze their independent association with mortality. Patients were stratified according to the diagnosis of diabetes.

**Results:**

Among patients without diabetes, mean BG bands between 80 and 140 mg/dl were independently associated with decreased risk of mortality, and mean BG bands >140 mg/dl, with increased risk of mortality. Among patients with diabetes, mean BG from 80 to 110 mg/dl was associated with increased risk of mortality and mean BG from 110 to 180 mg/dl with decreased risk of mortality. An effect of center was noted on the relation between mean BG and mortality. Hypoglycemia, defined as minimum BG <70 mg/dl, was independently associated with increased risk of mortality among patients with and without diabetes and increased glycemic variability, defined as CV >20%, was independently associated with increased risk of mortality only among patients without diabetes. Derangements of more than one domain of glycemic control had a cumulative association with mortality, especially for patients without diabetes.

**Conclusions:**

Although hyperglycemia, hypoglycemia, and increased glycemic variability is each independently associated with mortality in critically ill patients, diabetic status modulates these relations in clinically important ways. Our findings suggest that patients with diabetes may benefit from higher glucose target ranges than will those without diabetes. Additionally, hypoglycemia is independently associated with increased risk of mortality regardless of the patient's diabetic status, and increased glycemic variability is independently associated with increased risk of mortality among patients without diabetes.

See related commentary by Krinsley, http://ccforum.com/content/17/2/131

See related commentary by Finfer and Billot, http://ccforum.com/content/17/2/134

## Introduction

Stress-induced hyperglycemia during intensive care unit (ICU) admission has a strong and consistent relation with mortality [1-3]. Nevertheless, hyperglycemia in these populations of patients was not always treated with insulin infusion until the publication of a landmark single-center study in 2001 [4]. This trial demonstrated reductions in mortality when continuous intravenous insulin was used to achieve blood glucose (BG) from 80 to 110 mg/dl, compared with conventional therapy. Although these findings were corroborated in a large single-center cohort study [5], they were not confirmed by subsequent randomized trials [6-10].

One possible explanation for the divergent results among such trials may relate to the incidence of severe hypoglycemia sustained by patients in the interventional arms of randomized trials [6-11]. Data from observational [12-17] and interventional studies [4,6,11] demonstrated a strong and independent relation between hypoglycemia and mortality, even at milder thresholds, such as BG <70 mg/dl. Glycemic variability, not considered in the design or implementation of these trials, has also been independently associated with mortality in observational [18-24] and prospective [25] investigations. These findings have led to the emergence of the concept that three domains of glycemic control in the critically ill (hyperglycemia, hypoglycemia, and glycemic variability [26,27]) must be addressed to optimize glycemic control.

These factors, however, may not apply to all patients and, in particular, to those with the diagnosis of diabetes, presumably related to adaptive mechanisms developed in the setting of chronic hyperglycemia [28]. Observational cohort studies demonstrated that the relation between hyperglycemia and mortality is much stronger among patients without diabetes than in those with diabetes [3,29-31], and other observational data suggested that diabetes is not independently associated with increased risk of mortality and may actually have a modest protective effect [32-36].

The purpose of this study was to assess how diabetic status modulates the relation of the three domains of glycemic control to mortality in a large and diverse group of critically ill patients. We hypothesized that an association would exist between mortality and each of the three domains of glycemic control, but that a premorbid diagnosis of diabetes would attenuate the strength of these associations compared with those observed in patients without diabetes.

## Materials and methods

### Patient cohorts and clinical settings

Table 1 provides an overview of the nine different patient cohorts (Amsterdam (AM), Austin (AU), BayCare (BC), Birmingham (BI), Geelong (GE), Okayama (OK), Stamford (ST), Tufts (TU), and Vienna (VI)), the organizational structure of the ICUs, and the glycemic-control practices of the different centers.

**Table 1:** Overview of cohorts

	Amsterdam	Austin	BayCare	Birmingham	Geelong	Okayama	Stamford	Tufts	Vienna
Number of patients	1,660	1,172	19,738	5,529	4,562	3,601	5,032	2,290	1,440

Dates of admission to the ICU	1/09-12/09	10/09-3/11	7/07-6/10	4/09-3/12	9/05-12/10	4/08-6/11	10/05-6/11	3/10-5/12	2/01-3/09

Number and type of ICUs	Single 32-bed medical-surgical ICU of a university teaching hospital	Single 21-bed medical-surgical ICU of a university-affiliated teaching hospital	8 community-based hospitals, including 13 ICUs of mixed types, totaling 227 beds	Single 82-bed mixed (medical, surgical, cardiac, neurosciences, trauma, burns, and transplant) ICU of a university teaching hospital	Single 18-bed medical-surgical ICU of a university-affiliated teaching hospital	Two medical-surgical ICUs (total 22 beds) of a university-affiliated teaching hospital	Single 16-bed medical-surgical ICU of a university-affiliated teaching hospital	Single 10-bed surgical ICU of a university-affiliated teaching hospital	Single eight-bed medical ICU of a university hospital
Organizational details of centers	"Closed" format with intensivists supervising a team of critical care fellows, medical and surgical residents	Intensivist managed	All "Open" policy ICUs with mandate of critical care consult for all non-pure cardiac admission	Intensivist managed	Intensivist managed	Intensivist managed	Intensivist managed, with medical and surgical residents	Intensivist managed, with medical and surgical residents	Medical intensivist managed, with medical residents

Glycemic targets	90-144 mg/dl	108-180 mg/dl	70-110 mg/dl from 1/20/05-10/1/2008 then 80-150 mg/dl up to 10/1/2011 then 100-160 mg/dl	<180 mg/dl	a. Prior to April 2009: 4.1-8.0 m*M *(73.9-144.1 mg/dl) b. After April 2009: 7.1-10.0 m*M *(127.9-180.2 mg/dl)	<180 mg/dl	80-140 mg/dl from 10/1/05 to 1/10/07 80-125 mg/dl from 1/11/07 to 6/30/11	95-135 mg/dl since February 2002	<180 mg/dl to 06/03 80-110 mg/dl from 06/03-01/09 110-150 mg/dl from 01/09

Type of BG monitor	100% ABG analyzer (RapidLab 1200)	100% ABG analyzer	100% Accu-Chek Inform glucometers	100% ABG analyzer	100% ABG (Instrumentation Laboratory GEM 4000)	100% ABG analyzer	85% Accu-Chek Inform glucometers. 13% ABG analyzer 2% Central lab analyzer	98% Accu-check glucometer; 2% Central Lab analyzer	100% ABG analyzer

Source of blood	100% arterial	Venous or arterial blood	Capillary, venous, or arterial blood	98% arterial, 2% central venous	Arterial or venous blood	Venous or arterial blood	75% capillary 25% venous or arterial	70% Arterial, 23% central venous, and 2% capillary	100% arterial

Data acquisition	The blood glucose levels were extracted from the patient data-management system (MetaVision, iMDsoft, Israel). Other patient data were extracted from the National Intensive Care Evaluation (NICE) database, maintained by the NICE Foundation (reference: Arts D, de Keizer N, Scheffer GJ, de Jonge E. **Quality of data collected for severity-of-illness scores in the Dutch National Intensive Care Evaluation (NICE) registry**. *Intensive Care Med *2002, **28**:656-659.)	Glucose values captured automatically from arterial blood gas analyzers linked to hospital information system. Demographic and clinical data manually entered by trained data analysts into Australian National Adult Intensive Care database	ICUTracker Database linked to the hospital data systems	Glucose values captured automatically from arterial blood gas analyzers linked to hospital information system. Demographic and clinical data manually entered by trained data analysts into hospital database.	Glucose values captured automatically from arterial blood gas analyzers linked to hospital information system Demographic and clinical data manually entered by trained data analysts into Australian National Adult Intensive Care database	GAIA Database (Nihon Koden, Japan)	Comprehensive clinical database created in the ICU and linked to the hospital data systems	Glucostabilizer software program and ICUTracker Database.	Combination of clinical ward database (developed on the ICU) with BG-data retrieved from the ABG analyzer

### Outcomes

The primary end point for this analysis was all-cause hospital mortality, defined as death before hospital discharge.

### Definitions and statistical analysis

Patients were classified as having preexisting diabetes by documentation in their medical records. Disease severity was assessed by using APACHE II scores [37]. Descriptive statistics were calculated for all variables of interest. Continuous variables were summarized by using means and standard deviations, whereas categoric variables were summarized by using counts and percentages.

The primary outcome, mortality, was assessed in relation to the glycemic-control metric and control variables by using a logistic regression model adjusting for correlation among observations taken at the same center (that is, a generalized estimating equation (GEE) model. Three models were run, one for each glycemic measure: hyperglycemia, hypoglycemia, and glycemic variability. The models included a variable denoting diabetic status, the glycemic measure, and the key interaction term of diabetic status and glycemic measure. Each model controlled for mean BG, age, APACHE II score, mechanical ventilation, ICU length of stay (LOS), as well as adjusting for center effects. The models on hyperglycemia and glycemic variability also controlled for hypoglycemia (minimum BG <70 mg/dl). Each model was stratified by diagnostic category: medical or surgical. Patients admitted with trauma diagnoses were included in the surgical cohort.

Before analysis, the set of variables was assessed for the presence of multicollinearity. A tolerance statistic less than or equal to 0.4 was considered to indicate the presence of multicollinearity, and in such cases, only one member of a correlated set would be retained for the multivariable model.

The estimates of each model were presented by using odds ratios and their associated 95% confidence intervals. A Bonferroni correction was applied to account for multiple testing. As the greatest number of pairwise comparisons presented for a glycemic-control variable was 10, the standard *P *value of 0.05 was adjusted to 0.005 to denote statistical significance for all analyses.

Analyses were run by using SAS Version 9.2 (SAS Institute, Cary, NC, USA) and MedCalc V12.4.0.0 (Ostend, Belgium).

The institutional review boards of the different centers approved the investigation. The requirement for informed consent was waived because of the retrospective nature of the study and because the data were deidentified.

## Results

In Table 2a and b, we present the considerable heterogeneity in baseline clinical characteristics of the nondiabetic and diabetic cohorts in the nine different centers. The percentage of patients with diabetes in the different centers ranged from 14.0% (AM) to 38.6% (BC).

**Table 2:** Baseline characteristics, selected outcomes, and details of glycemic control

a. Nondiabetes patients										
	**ALL**	**Amsterdam**	**Austin**	**BayCare**	**Birmingham**	**Geelong**	**Okayama**	**Stamford**	**Tufts**	**Vienna**

**Number**	32,084	1,427	899	12,111	4,478	3,944	2,494	3,928	1,657	1,146

Age (years)	64 (50-77)	62 (48-72)	63 (49-75)	67 (52-80)	59 (43-70)	69 (57-77)	61 (39-73)	67 (51-80)	59 (46-73)	58 (46-68)

Male (%)	56.4	62.6	61.8	50.8	61.0	61.9	58.6	N/A	57.5	60.9

Patient type (%)										

Medical	56.8	37.0	55.6	81.2	30.8	35.3	32.1	52.0	70.1	80.9

Surgical	43.2	63.0	44.4	18.8	69.2	64.7	67.9	48.0	29.9	19.1

Ventilation (%)	41.3	84.2	69.5	27.6	26.3	69.8	53.5	37.2	39.4	77.8

APACHE II	19.0 (8.3)	19.0 (7.2)	16.2 (7.4)	23.4 (7.3)	13.8 (5.9)	16.2 (6.5)	13.5 (4.6)	15.6 (8.9)	15.5 (7.4)	16.0 (8.5)

ICU LOS	2.8 (1.6-5.2)	1.9 (1.0-3.9)	2.0 (1.1-4.0)	3.1 (2.0-5.1)	4.1 (2.2-8.0)	1.8 (1.0-2.9)	4 (3-7)	1.7 (0.9-3.5)	2.9 (1.8-5.4)	6 (3-11)

Mortality (%)	12.8	14.8	13.6	12.8	13.8	11.6	5.5	14.4	10.4	21.3

Glycemic control										

Mean BG (mg/dl)	129 (114-127)	135 (124-147)	130 (114-145)	128 (111-149)	139 (125-154)	131 (117-148)	137 (123-152)	121 (110-133)	123 (108-141)	119 (110-131)

**CV (%)**	17.7 (12.1-25.0)	17.7 (12.6-24.1)	16.0 (11.0-22.4)	19.0 (12.8-27.5)	17.5 (13.0-23.0)	18.5 (12.7-25.5)	13.5 (9.1-18.9)	18.9 (13.3-25.4)	18.6 (12.7-26.1)	21.4 (16.1-28.1)

Min BG <40	2.4	1.3	0.6	3.9	1.2	0.8	0.2	2.2	2.4	7.8

MIN BG 40-69	12.6	12.5	8.6	12.2	7.7	5.5	2.2	18.7	11.8	34.1

NO HYPO	85.0	86.2	90.8	84.9	91.1	93.7	97.6	79.1	85.8	58.1

Number BG	10 (5-21)	12 (7-28)	12 (7-23)	8 (4-17)	14 (7-31)	9 (6-16)	7 (4-18)	13 (7-29)	10 (5-21)	22 (11-49)

BG/24 hours^a^	4.5	7.0	5.7	3.5	3.9	5.5	2.8	9.0	4.3	4.5

b. Diabetes patients										

	**ALL**	**Amsterdam**	**Austin**	**BayCare**	**Birmingham**	**Geelong**	**Okayama**	**Stamford**	**Tufts**	**Vienna**

Number	12,880	233	278	7,626	1,051	618	1,043	1,104	633	294

Age (years)	68 (59-79)	66 (60-75)	67 (59-75)	70 (59-79)	65 (56-73)	66 (57-74)	67 (57-75)	70 (61-80)	69 (57-77)	65 (56-74)

Male (%)	56.4	67.4	64.7	53.1	64.3	59.3	65.3	N/A	56.2	61.6

Patient type (%)										

Medical	70.2	39.5	54.0	85.0	38.4	45.1	28.1	63.0	75.3	77.9

Surgical	29.8	60.5	46.0	15.0	61.6	54.9	71.9	37.0	24.7	22.1

Ventilation (%)	30.9	83.7	73.0	23.1	17.6	58.0	48.1	39.9	38.5	77.8

APACHE II	21.9 (8.1)	21.1 (7.4)	17.8 (7.0)	24.4 (7.3)	16.0 (5.7)	16.7 (7.4)	15.1 (4.4)	18.5 (8.8)	17.0 (7.8)	16.5 (8.2)

ICU LOS	2.8 (1.6-5.0)	1.9 (1.0-3.9)	2.0 (1.1-4.4)	2.8 (1.7-4.8)	4.1 (2.3-8.0)	1.8 (1.0-3.5)	4 (3-7)	1.9 (1.0-4.2)	2.5 (1.5-5.0)	6 (3-11)

Mortality (%)	13.3	15.5	10.8	12.4	17.7	11.9	8.8	16.7	16.0	22.1

Glycemic control										

Mean BG (mg/dl)	153 (129-182)	152 (139-169)	156 (142-172)	154 (128-188)	166 (145-189)	152 (124-180)	153 (135-175)	137 (122-153)	157 (129-194)	135 (121-155)

CV (%)	25.5 (17.0-36.4)	26.3 (18.5-33.2)	23.7 (16.9-31.2)	27.1 (18.7-38.5)	24.7 (17.9-33.4)	27.3 (20.4-36.6)	16.2 (11.0-23.9)	28.5 (21.2-38.5)	26.1 (17.9-36.8)	30.7 (22.8-38.6)

Min BG <40	5.4	4.3	1.4	7.1	3.4	3.1	1.0	6.1	4.7	13.3

Min BG 40-69	19.6	19.4	14.4	19.1	10.5	23.3	3.6	31.1	15.2	38.8

No hypo	75.0	76.6	84.2	73.8	86.1	73.6	95.4	62.8	80.1	47.9

Number BG	12 (6-26)	14 (8-31)	13 (9-29)	11 (6-23)	16 (8-32)	11 (7-20)	9 (4-21)	17 (8-42)	12 (6-30)	22 (12-54)

BG/24 hours^a^	5.5	8.2	6.4	5.3	4.1	5.6	2.9	10.6	7.5	4.9

a. Okayama cohort: Age, Patient type, APACHE II score, Ventilation (%), ICU LOS based on subset of 260 patients. Birmingham cohort: APACHE II score based on subset of 483 patients. b. Okayama cohort: Age, Patient type, APACHE II score, Ventilation (%), ICU LOS based on subset of 837 patients. Birmingham cohort: APACHE II score based on subset of 2,516 patients. ^a^Calculated as mean BG values/mean ICU LOS.

### Glycemic control

Patients with diabetes had higher mean BG, higher CV, and higher rates of hypoglycemia than did patients without diabetes. The nine centers demonstrated considerable variation in the frequency of BG monitoring as well as in the intensity of glycemic control, as reflected by mean BG.

### Three domains of glycemic control: unadjusted mortality data, nine centers

#### Mean BG

**Figure 1:**
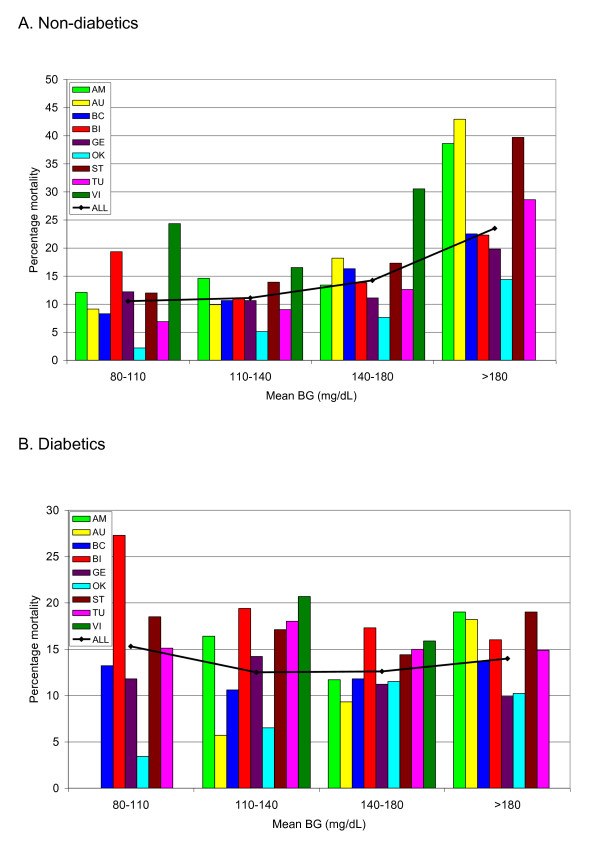
**Mean blood glucose (BG) and mortality**. The relation of mean BG (milligrams per deciliter) during ICU stay to mortality in those without **(A) **and those with diabetes **(B)**, for each of the nine cohorts as well as the entire population.

Figure 1A and 1B displays the unadjusted relation between mean BG and mortality for the nine centers. Additional file 1, Table S1 in the online supplement delineates the number of patients in each "band" of mean BG, as well as their mean (95% confidence interval (CI)) mortality. Among patients without diabetes, mortality was lowest when mean BG was 80 to 110 and 110 and 140 mg/dl and increased at higher levels. The mortality rate of the 200 patients with mean BG <80 mg/dl (0.62% of the total of 32,084 patients without diabetes) was 47.0%. Among patients with diabetes, the shape of the relation between mean BG and mortality was characterized as a shallow trough, with modestly higher mortality in the aggregate with mean BG 80 to 110 and >180 mg/dl than with mean BG in the 110- to 180-mg/dl range. The mortality rate of the 71 patients with mean BG <80 mg/dl (0.55% of the total of 12,880 patients with diabetes) was 42.3%.

#### Hypoglycemia

**Figure 2:**
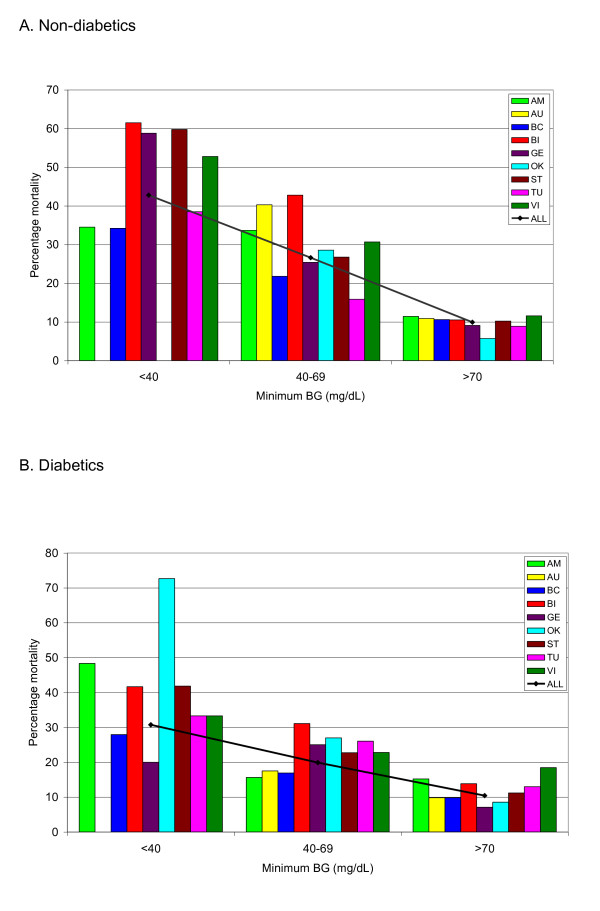
**Minimum BG and mortality**. The relation of minimum BG (milligrams per deciliter) during ICU to mortality in nondiabetes **(A) **and diabetes **(B) **patients, for each of the nine cohorts as well as the entire population. Cohorts with fewer than 20 patients in a particular "band" are not reported.

Figure 2A and 2B illustrates the unadjusted relation between hypoglycemia and mortality. Hypoglycemia was associated with increased mortality in patients with diabetes as well as in patients without diabetes, although the relation was stronger among patients without diabetes.

#### Glycemic variability

**Figure 3:**
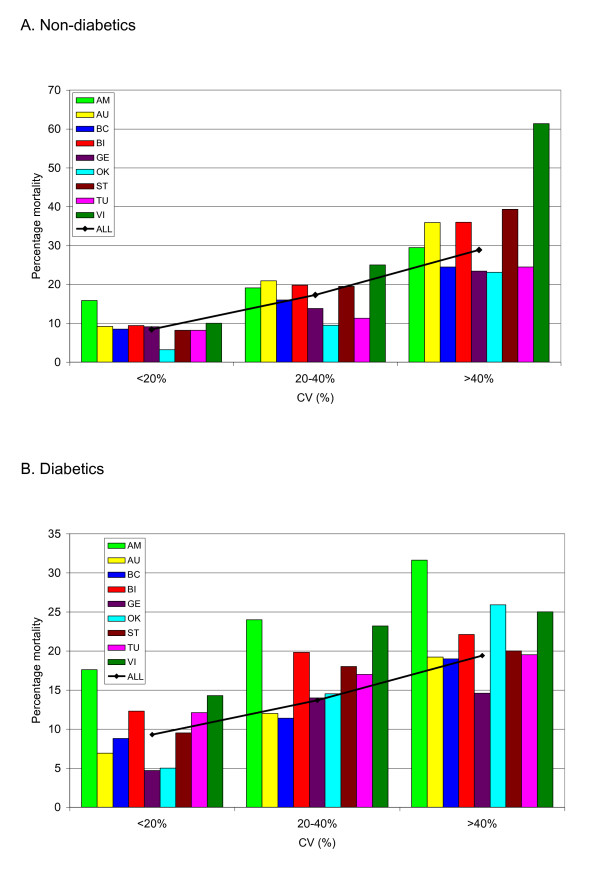
**Coefficient of variation and mortality**. The relationp of coefficient of variation (%) to mortality in nondiabetes **(A) **and diabetes **(B) **patients for each of the nine cohorts as well as the entire population. Cohorts with fewer than 20 patients in a particular "band" are not reported.

Figure 3A and 3B displays the unadjusted relation between CV and mortality. Among patients without diabetes, the relation between increasing CV and increasing mortality was steep, with more than a threefold higher mortality among the entire cohort with CV >40% compared with those with CV <20%. This relation was similar, albeit attenuated, among patients with diabetes.

### Cumulative derangements in the three domains of glycemic control and their association with mortality

**Figure 4:**
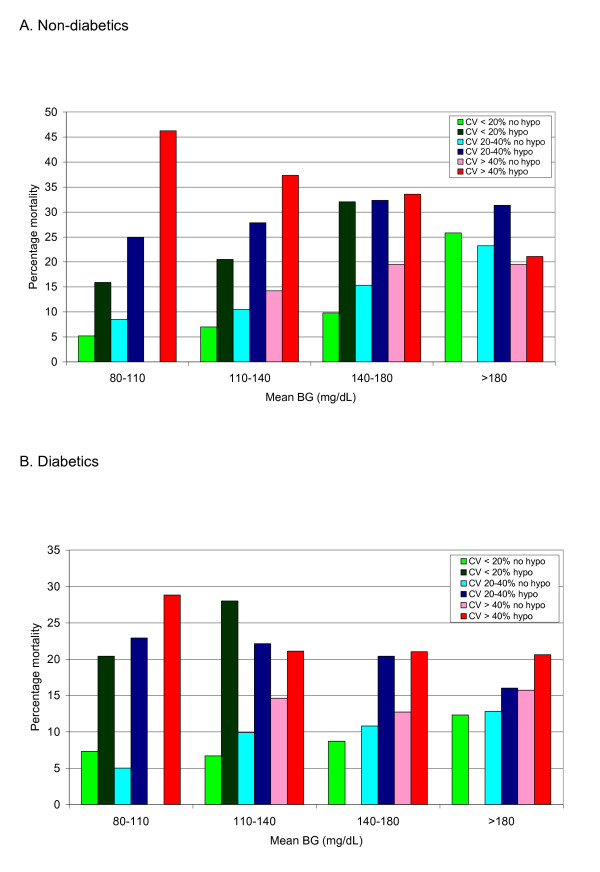
**Cumulative derangements of three domains**. The relation of cumulative derangements of the three domains of glycemic control to mortality in nondiabetes **(A) **and diabetes **(B) **patients. Patients are stratified first by mean BG during ICU stay, then by increasing coefficient of variation (CV), and then by the presence or absence of hypoglycemia, defined as minimum BG <70 mg/dl during ICU stay. "Bands" with fewer than 20 patients are not reported.

Figure 4A and 4B illustrates the cumulative impact of derangements in the three domains of glycemic control. Among patients without diabetes who had mean BG between 80 and 110, 110 and 140, and 140 and 180 mg/dl, increasing CV and the occurrence of hypoglycemia were associated with increased mortality, and their effect was cumulative. Among patients without diabetes with mean BG >180 mg/dl, no incremental impact was found of additional derangements of glycemic control. Among patients with diabetes, hypoglycemia was consistently associated with increased mortality, but mean BG and CV did not have a consistent, cumulative impact on mortality.

### Multivariable analysis

**Figure 5:**
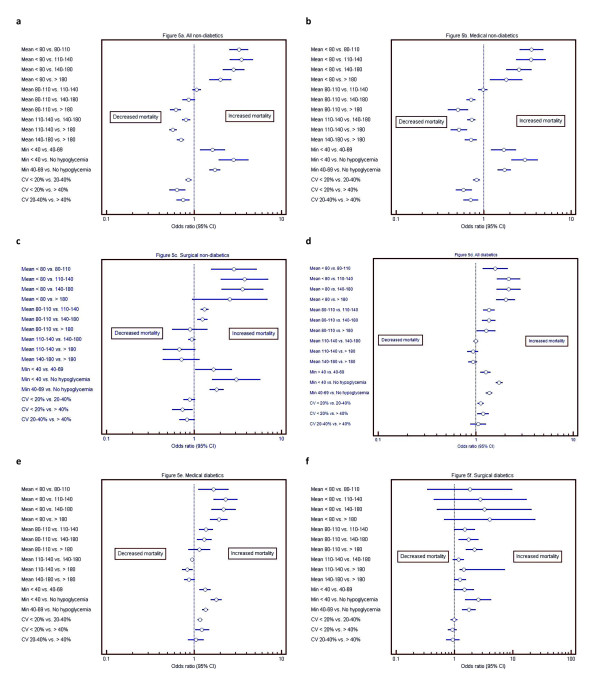
**Forest plots of bands of the independent association of mean BG, hypoglycemia, and coefficient of variation to mortality, for diabetes and nondiabetes patients**. This figure illustrates the independent association of mean BG, hypoglycemia, and coefficient of variation to mortality, for diabetes and nondiabetes patients, including stratification based on medical versus surgical status. Pair-wise comparisons of odds ratio (95% CI) for each domain of glycemic control are presented.

Figure 5A through F displays the results of multivariable analysis, assessing the independent association of bands within each domain with mortality.

#### Mean BG

An effect of center was seen on the relation between mean BG and mortality. Among patients without diabetes, mean BG of 110 to 140 mg/dl was independently associated with reduced risk of mortality compared with mean BG of 140 to 180 and >180 mg/dl, and similar risk compared with mean BG of 80 to 110 mg/dl.

The medical and surgical patients demonstrated different patterns. Among medical patients, bands of mean BG of 80- to 140-mg/dl range were independently associated with the lowest risk of mortality, with increased risk of mortality at higher bands. In contrast, among surgical patients, a mean BG of 80 to 110 mg/dl was independently associated with increased risk of mortality compared with bands of mean BG of 110 to 180 mg/dl.

The relation of mean BG to mortality was somewhat different among patients with diabetes. Among the entire cohort of patients with diabetes, as well as for both medical and surgical subpopulations, mean BG of 80 to 110 mg/dl was independently associated with increased risk of mortality compared with the bands of mean BG of 110 to 180 mg/dl, those with mean BG of 110 to 140, 140 to 180, and <180 mg/dl had a reduced risk of mortality.

#### Hypoglycemia

Severe (minimum BG <40 mg/dl) and mild to moderate (BG of 40 to 69 mg/dl) hypoglycemia were independently associated with increased risk of mortality, for the entire cohort, as well as for the medical and surgical subpopulations.

#### Glycemic variability

Among patients without diabetes, low glycemic variability (CV <20%) was independently associated with decreased risk of mortality compared with bands of CV of 20% to 40% and >40% for the entire cohort; this relation was more robust in medical patients than in surgical patients. However, among patients with diabetes, multivariable analysis demonstrated that increased CV was not independently associated with increased risk of mortality.

#### Diabetes

**Figure 6:**
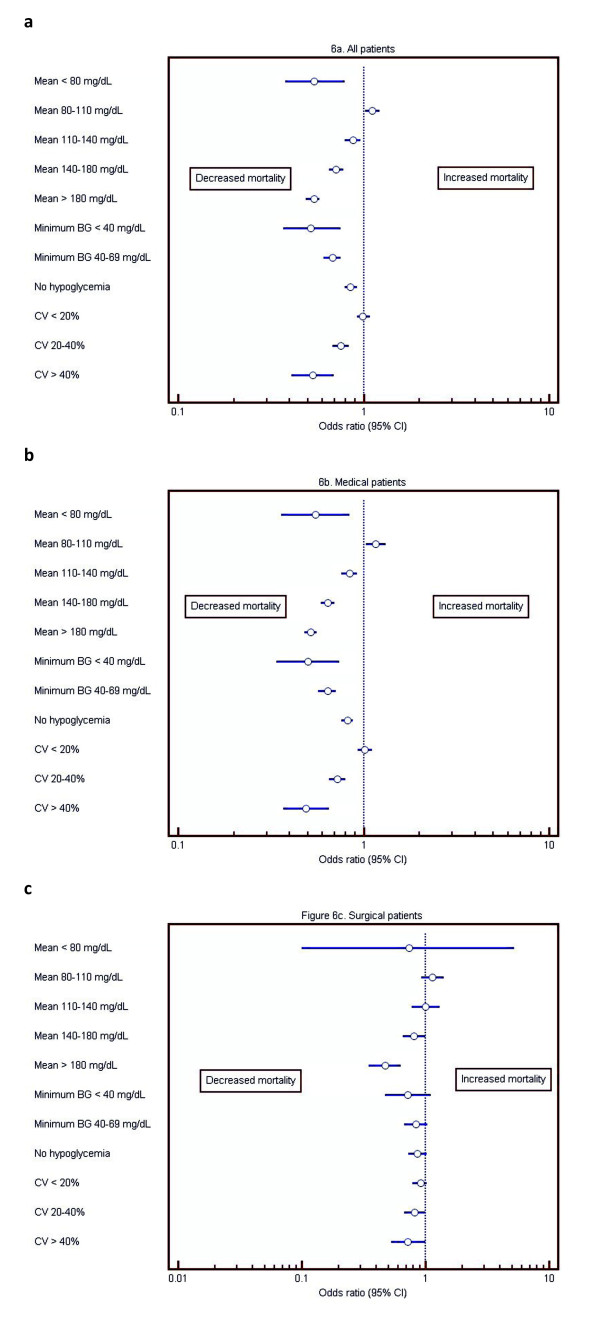
**Forest plots describing the independent association of diabetes with mortality, for each of the three domains of glycemic control**. This figure illustrate the independent association of diabetic status with mortality associated with each of the three domains of glycemic control. For example, Figure 6a demonstrates that, among patients with mean BG 80 to 110 mg/dl, diabetes was independently associated with increased risk of mortality, but among patients with mean BG of 110 to 140 mg/dl, diabetes was independently associated with decreased risk of mortality.

Diabetes was independently associated with decreased risk of mortality for the entire cohort (OR (95% CI)) 0.93 (0.87 to 0.97); *P *= 0.0030. Figure 6 displays the results of multivariable analysis assessing the independent association of diabetes with mortality, stratified by individual bands of the three domains of glycemic control. Among patients with mean BG of 80 to 110 mg/dl, diabetes was independently associated with increased risk of mortality for the entire cohort and the medical subgroup of <80 to >110 mg/dl. However, for all other bands of mean BG, diabetes was associated with decreased risk of mortality for the entire cohort and the medical subgroup. Diabetes was not independently associated with mortality in the surgical subgroup. Similarly, among the entire cohort with hypoglycemia and in the medical subgroup with hypoglycemia, diabetes was independently associated with decreased mortality; diabetes was not independently associated with mortality among hypoglycemic surgical patients.

Finally, diabetes was independently associated with decreased mortality among the entire cohort and both subgroups in patients with increased glycemic variability, defined as CV >20%.

## Discussion

### Salient findings

This multicenter investigation demonstrates clinically important differences between critically ill patients with diabetes and patients without diabetes in regard to the relation between the three domains of glycemic control and mortality. Among patients without diabetes, the lowest mortality occurred in patients with mean BG of 80 to 140 mg/dl. In contrast, among patients with diabetes, mean BG of 80 to 110 mg/dl was independently associated with increased risk of mortality compared with patients with a mean BG of 110 to 140, 140 to 180, and even >180 mg/dl. Hypoglycemia was independently associated with increased risk of mortality among patients with diabetes as well as among those without diabetes. Increased glycemic variability (CV >20%), however, was independently associated with increased risk of mortality among patients without diabetes but not among patients with diabetes. Derangements in more than one domain of glycemic control were associated with cumulative increase in mortality among nondiabetes patients but not among patients with diabetes. Finally, for the entire cohort of 44,964 patients, diabetes was independently associated with decreased risk of mortality.

### Relation to prior literature

Hyperglycemia is associated with increased mortality in the critically ill [2,3,14,29-31]. Increments of mean BG levels above 80 mg/dl are clearly associated with increasing mortality among patients without diabetes. In contrast, a blunted relation exists between increasing mean BG levels above 80 mg/dl and mortality among patients with diabetes [3,29-31]. It is likely that changes in glycemic-control practice over time have altered the observed relation between mean BG and mortality. The current investigation reflects contemporary practice; all patients were admitted to ICUs practicing at least "moderate" glycemic control; the range of mean BG values of the patients without diabetes in the different centers (119 to 137 mg/dl) contrasts sharply with the mean morning BG of the patients in the control arm of the first Leuven trial of IIT (153 mg/dl) [4].

Hypoglycemia was the second of the three domains to be associated with increased risk of mortality in critically ill patients. Although most of the literature has described an independent association of severe hypoglycemia (minimum BG <40 mg/dl) with mortality [12-15,22], recent observational studies [16,17] and prospective trial data [11] have identified mild hypoglycemia (minimum BG <70 mg/dl) as being independently associated with increased risk of mortality. Our findings confirm these observations for patients with and without diabetes.

Glycemic variability was the third of the three domains to be independently associated with mortality in the critically ill [18-25]. One observational study suggested that glycemic variability was independently associated with mortality only among critically ill patients without diabetes [24]; our study confirms these findings.

Finally, the independent impact of diabetic status, without reference to glycemic control, on the mortality of critically ill patients has been the subject of recent observational studies that concluded that patients with diabetes did not experience higher mortality, and diabetes may, in fact, be protective [30-36]. We demonstrated here that diabetes is independently associated with decreased risk of mortality.

### Strengths and weaknesses

The clearest strength of this study is its size. The 44,964 patients include patients admitted with a large array of medical, surgical, and trauma diagnoses, treated with a variety of glycemic-control protocols, substantially enhancing the generalizability of the investigation. Moreover, this is a modern cohort of patients treated in an era characterized by attention to glycemic control. Each of the nine centers maintained a robust database characterized by prospective data collection, creating an additional important strength of this investigation: the breadth of demographic, clinical outcome, and glycemic data available for analysis. The absence of information about insulin therapy is an important limitation. It is likely that important differences exist between insulin-treated and insulin-naive patients regarding the relation of the three domains of glycemic control to mortality.

Another potential limitation is that the identification of diabetic status was made on clinical grounds, based on all information available at the time of ICU admission. It is likely that some patients designated as without diabetes may actually have had diabetes; HgbA1c levels were not obtained routinely, and, of course, glucose-tolerance testing could not be performed. Furthermore, we are unable to determine whether the diabetes patients were categorized as type I or type II. Although most were likely type II, important differences may exist between the two groups in their response to derangements in the domains of glycemic control. Additionally, we cannot provide details of nutritional therapy and cannot therefore analyze the interactions among glycemic control, nutritional therapy, and insulin treatment of hyperglycemia. Moreover, many of the glycemia data from several of the centers included in this study were derived from capillary blood measured on point-of-care devices, a method associated with increased analytic inaccuracy [38-41]. Nevertheless, any degree of measurement imprecision would only serve to dampen the observed relations between glycemia and diabetic status.

Finally, we acknowledge that the observational nature of this investigation mandates that its conclusions must be considered to be hypothesis generating, rather than proof of causality. Nevertheless, it would be unethical to randomize patients to induced hyperglycemia, hypoglycemia, or increased glycemic variability.

### Biological plausibility

Considerable evidence suggests that diabetes may alter the relation between glycemia and mortality in critically ill patients [28]. Diabetes patients may develop a tolerance to hyperglycemia, and a moderate degree of hyperglycemia that might exert toxicity in a patient without diabetes may be well tolerated in a patient with diabetes. This may explain the strong relation seen between increasing mean BG levels and mortality in patients without diabetes, detailed in several large observational studies, but not among those with diabetes [3,29-31,36,42]. In a recent study [43], diabetes patients with poor preadmission glycemic control, reflected by high HgbA1c levels, had higher mortality when mean BG was tightly controlled during ICU stay compared with patients with high premorbid HgbA1c levels who had a higher mean BG during ICU stay. These intriguing data parallel the results of large interventional studies in outpatient populations with type II diabetes [44,45]. An extensive body of literature has explored the physiological basis of the deleterious impact of hypoglycemia [46-51] demonstrated in interventional [4,6,11,25] and observational [12-17] studies; none of these has focused explicitly on the different impact that hypoglycemia may exert on patients with diabetes compared with those without diabetes. Similarly, although various physiological mechanisms underlying the harmful effect of increased glycemic variability detailed in interventional [4,6,25] and observational [18-24] studies have been proposed [52-56], the reasons that glycemic variability has no or a muted independent association with risk of mortality in patients with diabetes compared with the striking relation seen in patients without diabetes requires further clarification.

### Clinical implications

The central findings of the current investigation have important implications for the care of critically ill patients. Hyperglycemia does not have the same association with mortality among critically ill patients without diabetes compared with those with diabetes. The euglycemic range was independently associated with the lowest risk of mortality among patients without diabetes but with higher mortality among patients with diabetes. Additionally, important differences were noted when comparing medical and surgical populations. These findings call into question the "one size fits all" strategy for glycemic control of critically ill patients. It may be most appropriate to establish lower glycemic target ranges for medical patients without diabetes than for patients with diabetes or for surgical patients without diabetes.

In addition, our observations call into question the appropriateness of recently published glycemic-control guidelines that recommend a glycemic target range of 140 to 180 mg/dl [57] or 140 to 200 mg/dl [58] for all critically ill patients. Furthermore, premorbid glycemic control in diabetes may have an important impact on the consequences of glycemic control in the ICU [43]. The optimal glycemic-control protocol may result not only from stratifying patients by diabetic status, but also by additionally stratifying patients with diabetes based on the degree of preadmission glycemic control. In contrast, the deleterious association of hypoglycemia with mortality, even at threshold levels of <70 mg/dl, was observed in patients with diabetes and in those without diabetes. Because hypoglycemia can never be the subject of a randomized trial, the data from this investigation, when combined with the findings from previous interventional [4,6,10,11,25] and observational [12-17] studies, provide the strongest evidence basis for the goal of avoiding hypoglycemia in all critically ill patients.

Finally, increased glycemic variability, defined as CV >20%, was identified in the current study as having a strong independent association with increased risk of mortality in patients without diabetes. These data provide strong impetus for the creation of insulin-dosing strategies and the development of new technologies [59] for accurate continuous or near-continuous BG monitoring, with the goal of reducing glycemic variability in critically ill patients. Further investigation should stratify patient outcomes by specific admitting diagnosis; important differences may be found within the broad medical and surgical categories that the current investigation was underpowered to assess.

The design of future trials of IIT should include consideration of all three domains of glycemic control as well as recognition of the differences in their association with mortality based on premorbid diabetes status.

## Conclusions

This large international cohort study evaluated the relation of diabetic status to the association of hyperglycemia, hypoglycemia, and increased glycemic variability in a heterogeneous population of critically ill patients. We found that diabetic status modulates the relation between the three domains of glycemic control and mortality in clinically important ways. Our findings suggest that patients with diabetes may benefit from higher glucose target ranges than those without diabetes. Additionally, hypoglycemia is independently associated with increased risk of mortality, regardless of the patient's diabetic status, and increased glycemic variability is independently associated with increased risk of mortality among patients without diabetes. These findings may inform the implementation of glycemic-control protocols in the intensive care unit, as well as for the design of future interventional trials of intensive monitoring and treatment of dysglycemia in the critically ill.

## Key messages

• Diabetic status modulates the relation between the three domains of glycemic control (hyperglycemia, hypoglycemia, and glycemic variability) and mortality in critically ill patients in clinically important ways.

• The range of mean BG from 80 to 140 mg/dl is associated with the lowest severity adjusted mortality among nondiabetes patients. In contrast, among those with diabetes, a mean BG of 80 to 110 mg/dl is associated with higher mortality risk than is the range of 110 to 180 mg/dl.

• A single episode of hypoglycemia (BG <70 mg/dl) is independently associated with increased risk of mortality among those without as well as those with diabetes.

• Increased glycemic variability, defined as CV >20%, is independently associated with increased risk of mortality among those without, but not among those with diabetes.

## Abbreviations

ABG: arterial blood gas; APACHE: acute physiology and chronic health evaluation; BG: blood glucose; CV: coefficient of variation; DM: diabetes mellitus; ICU: intensive care unit; IIT: intensive insulin therapy; LOS: length of stay; OR: odds ratio.

Participating centers in this investigation: AM: Amsterdam; AU: Austin; BC: BayCare; BI: Birmingham; GE: Geelong; OK: Okayama; ST: Stamford; TU: Tufts; VI: Vienna.

## Competing interests

Dr. Krinsley reported receiving consultant fees from Medtronic Inc., Edwards Life Sciences, Roche Diagnostics, OptiScan Biomedical, and Alere and research support from OptiScan Biomedical. He also received royalty payments for sales of ICU Tracker. Dr. Amin reported receiving speaker fees from BioMerueux. Ms. Maurer works as a consultant for Alere, the distributor of ICU Tracker. Dr. Schultz reported receiving consultant fees from Medtronic Inc., GlySure Ltd., and Roche Diagnostics, and research support from Medtronic Inc. and OptiScan Biomedical. Dr. van Hooijdonk reported consultant fees from Medtronic Inc. and GlySure Ltd., and research support from Medtronic Inc. and OptiScan Biomedical. Dr. Annane reported serving on advisory board meetings for Edwards Life Sciences but did not receive compensation. Dr. Nasraway reported receiving consultant fees from GlySure Ltd., OptiScan Biomedical, and Edwards Life Sciences, and consulting fees and stock options from Echo Therapeutics. Dr. Holzinger reported receiving consultant fees from Medtronic Inc. and speaker fees from NovoNordisk. Dr. Preiser reported receiving consultant fees from Medtronic Inc., Edwards Life Sciences, and OptiScan Biomedical.

Dr. Egi, Dr. Kiss, Dr. Amin, Dr. Schuetz, Dr. Kiyoshi, Dr. Mackenzie, Dr. Stow, Ms. Holewinski, Dr. Vincent, and Dr. Bellomo reported no relevant interests.

## Authors' contributions

JK conceived of the study concept and design, wrote the draft of the manuscript, had full access to all of the data in the study, and takes responsibility for the integrity of the data and the accuracy of the data analysis. JK, ME, DA, PS, PM MS, RvH, KM, IM, PS, SN, SH, UH, and RM participated in data acquisition. JK, ME, MS, JP, and RB performed analysis and interpretation of the data. ME, DA, PS, MS, IA, DA, SN, RvH, JP, UH, JP, JV, and RB performed critical revision of the manuscript for important intellectual content. AK and JK completed the statistical analysis. All of the authors read and approved the final draft of the manuscript.

## Supplementary Material

Additional file 1 **Table S1**. Mortality (percentage, 95% CI) and number of patients for individual cohorts, nondiabetes, and diabetes patients, for each of the three domains of glycemic control. This file contains data detailing the number of patients from each of the nine centers, their mortality percentage, and the 95% CI of this percentage, stratified by diabetic status, for each of the "bands" of the three domains of glycemic control described in the manuscript. Click here for file
