# Supplementary material for: Diabetic status and the relation of the three domains of glycemic control to mortality in critically ill patients: an international multicenter cohort study
Source: Crit Care. 2013 Mar 1;17(2):R37. doi: 10.1186/cc12547 (PMC3733432; doi:10.1186/cc12547)
Supplement: Additional file 1 — Table S1. Mortality (percentage, 95% CI) and number of patients for individual cohorts, nondiabetes, and diabetes patients, for each of the three domains of glycemic control. This file contains data detailing the number of patients from each of the nine centers, their mortality percentage, and the 95% CI of this percentage, stratified by diabetic status, for each of the "bands" of the three domains of glycemic control described in the manuscript. [file cc12547-S1.DOC]

Table S1

Mortality (percentage, 95% CI) and number of patients for individual cohorts, non-diabetics and diabetics, for each of the 3 domains of glycemic control

Mean BG (mg/dL)

|  | 80-110 | 110-140 | 140-180 | >180 |
| --- | --- | --- | --- | --- |
| **NON** |  |  |  |  |
| AM | 12.1  (6.3-17.9)  124 | 14.6  (12.1-17.2)  758 | 13.4  (10.4-16.5)  491 | 38.6  (23.7-53.6)  44 |
| AU | 9.1  (4.5-13.7)  154 | 9.9  (7.1-12.8)  423 | 18.3  (13.7-22.8)  285 | 42.9  (23.3-62.4)  28 |
| BC | 8.3  (7.2-9.3)  2,678 | 10.6  (9.7-11.4)  5,232 | 16.3  (15.0-17.6)  3,045 | 22.5  (20.0-25.0)  1,064 |
| BI | 19.3  (15.5-23.2)  409 | 11.1  (9.6-12.5)  1,861 | 13.8  (12.2-15.3)  1,963 | 22.3  (16.8-27.8)  224 |
| GE | 12.2  (9.3-15.1)  500 | 10.6  (9.3-12.0)  2,012 | 11.1  (9.4-12.9)  1,184 | 19.8  (14.3-25.3)  207 |
| OK | 2.2  (0.2-4.1)  231 | 5.1  (3.8-6.4)  1,130 | 7.6  (6.0-9.2)  1,015 | 14.4  (7.8-21.1)  111 |
| ST | 12.0  (9.9-14.0)  953 | 13.9  (12.5-15.3)  2,360 | 17.3  (14.0-20.5)  533 | 39.7  (26.7-52.6)  58 |
| TU | 6.9  (4.5-9.2)  437 | 9.0  (7.0-11.0)  777 | 12.6  (9.0-16.2)  333 | 28.6  (19.5-37.7)  98 |
| VI | 24.3  (19.1-30.0)  263 | 16.5  (13.8-19.3)  703 | 30.5  (23.2-37.9)  154 | 58.3  (25.6-91.1)  12 |
| ALL | 10.5  (9.7-11.3)  5,749 | 11.1  (10.6-11.6)  15,256 | 14.2  (13.4-14.9)  9,003 | 23.5  (21.5-25.4)  1,846 |
|  |  |  |  |  |
| **DM** |  |  |  |  |
| AM | 42.9  NA  7 | 16.4  (6.3-26.5)  55 | 11.7  (6.1-17.4)  128 | 19.1  (6.7-31.4)  42 |
| AU | 13.3  NA  15 | 5.7  NA  35 | 9.3  (5.0-13.5)  183 | 18.2  (6.3-30.0)  44 |
| BC | 13.3  (10.7-15.8)  687 | 10.6  (9.2-11.9)  2,035 | 11.8  (10.6-13.1)  2,603 | 13.7  (12.3-15.2)  2,251 |
| BI | 27.3  (13.6-41.0)  44 | 19.4  (13.2-25.6)  160 | 17.3  (13.9-20.7)  480 | 16.0  (12.2-19.8)  363 |
| GE | 11.8  (2.6-20.9)  51 | 14.2  (9.3-19.1)  197 | 11.2  (7.0-15.5)  214 | 9.9  (5.1-14.7)  152 |
| OK | 3.4  NA  29 | 6.5  (3.6-9.5)  276 | 11.5  (8.7-14.2)  523 | 10.2  (6.1-14.3)  215 |
| ST | 18.5  (11.7-25.2)  130 | 17.1  (13.8-20.5)  491 | 14.4  (10.8-17.9)  376 | 19.0  (11.2-26.8)  100 |
| TU | 15.1  (5.1-25.1)  53 | 18.0  (12.0-24.0)  161 | 15.0 (10.2-19.9)  213 | 14.9  (9.9-19.8)  202 |
| VI | 57.9  (33.5-82.3)  19 | 20.7  (14.0-27.4)  145 | 15.9  (8.8-22.9)  107 | 30.0  (8.0-52.0)  20 |
| ALL | 15.3  (13.1-17.5)  1,035 | 12.6  (11.5-13.6)  3,555 | 12.6  (11.7-13.6)  4,827 | 14.0  (12.9-15.2)  3,389 |

2. Minimum BG (mg/dL)

|  | <40 | 40-69 | >70 |
| --- | --- | --- | --- |
| **NON** |  |  |  |
| AM | 61.1  (36.2-86.1)  18 | 33.7  (26.7-40.7)  178 | 11.4  (9.6-13.2)  1,231 |
| AU | 40.0  NA  5 | 40.3  (29.1-51.5)  77 | 11.0  (8.8-13.1)  813 |
| BC | 34.2  (30.0-38.7)  424 | 21.8  (19.7-23.9)  1,527 | 10.6  (10.0-11.2)  10,160 |
| BI | 71.5  (47.9-75.2)  52 | 42.8  (37.7-47.8)  374 | 10.5  (9.5-11.4)  4,052 |
| GE | 60.0  (41.4-78.6)  30 | 25.4  (21.6-29.2)  512 | 9.1  (8.2-10.1)  3,376 |
| OK | 25.0  (4.2-45.8)  20 | 28.6  (16.4-40.1)  56 | 5.7  (4.7-6.6)  2,418 |
| ST | 59.8  (49.3-70.1)  87 | 26.8  (26.3-30.0)  736 | 10.2  (9.2-11.3)  3,105 |
| TU | 38.5  (22.5-54.4)  39 | 15.9  (10.7-21.1)  195 | 8.9  (7.4-10.4)  1,423 |
| VI | 52.8  (42.2-63.4)  89 | 30.7  (26.1-35.3)  391 | 11.6  (9.1-14.0)  666 |
| ALL | 42.8  (39.3-46.3)  764 | 26.6  (25.3-28.0)  4,046 | 9.9  (9.5-10.3)  27,244 |
|  |  |  |  |
| **DM** |  |  |  |
| AM | 20.0  NA  10 | 15.6  (4.5-26.6)  45 | 15.2  (9.8-20.5)  178 |
| AU | 0.0  NA  4 | 17.5  (5.2-29.8)  40 | 9.8  (6.0-13.7)  234 |
| BC | 27.9  (23.9-32.0)  476 | 16.9  (15.1-18.8)  1,523 | 9.8  (9.0-10.6)  5,627 |
| BI | 41.7  (24.8-58.6)  36 | 31.1  (24.3-37.9)  180 | 13.8  (11.4-16.1)  835 |
| GE | 26.3  (4.5-48.1)  19 | 25.0  (17.8-32.2)  144 | 7.1  (4.7-9.4)  453 |
| OK | 53.9  (22.5-85.2)  13 | 27.0  (12.0-42.0)  37 | 8.5  (6.7-10.2)  993 |
| ST | 41.8  (30.0-53.9  67 | 22.7  (18.3-27.2)  343 | 11.2  (8.9-13.6)  694 |
| TU | 33.3  (15.4-51.2) 30 | 26.0  (17.1-35.0)  96 | 13.0 (10.1-16.0)  507 |
| VI | 33.3  (17.9-48.8)  39 | 22.8  (15.0-30.6)  114 | 18.4  (12.0-24.9)  141 |
| ALL | 30.7  (27.3-34.1)  694 | 19.9  (18.4-21.5)  2,522 | 10.4  (9.8-11.0)  9,662 |

3. CV (%)

|  | <20% | 20-40% | >40% |
| --- | --- | --- | --- |
| **NON** |  |  |  |
| AM | 9.2  (7.3-11.1)  857 | 21.9  (18.3-25.4)  521 | 36.7  (22.7-50.7)  49 |
| AU | 9.1  (6.8-11.4)  604 | 20.7  (15.7-25.7)  256 | 40.0  (22.9-57.1)  35 |
| BC | 8.5  (7.9-9.2)  6,539 | 16.1  (15.0-17.1)  4,375 | 24.5  (22.0-26.9)  1,197 |
| BI | 9.4  (8.4-10.5)  2,819 | 19.8  (17.8-21.8)  1,520 | 36.0  (27.9-44.1)  139 |
| GE | 9.1  (7.9-10.3)  2,207 | 13.8  (12.1-15.6)  1,505 | 23.3  (17.5-29.1)  206 |
| OK | 5.6  (4.7-6.6)  2,325 | 14.7  (9.2-20.2)  163 | 50.0  NA  6 |
| ST | 8.2  (7.0-9.3)  2,157 | 19.5  (17.5-21.5)  1,542 | 39.3  (32.9-45.7)  229 |
| TU | 8.2  (6.4-10.0)  926 | 11.3  (8.8-13.8)  1,621 | 24.6  (16.4-32.7)  110 |
| VI | 10.0  (7.3-12.6)  482 | 25.0 (21.4-28.5)  581 | 61.5  (50.8-72.1)  83 |
| ALL | 8.4  (8.0-8.8)  18,916 | 17.3  (16.6-18.0)  11,084 | 28.9  (27.0-30.9)  2,054 |
|  |  |  |  |
| **DM** |  |  |  |
| AM | 15.8  (7.4-24.2)  76 | 13.9  (7.8-19.9)  130 | 22.2  (5.5-39.0)  27 |
| AU | 6.9  (1.9-11.9)  102 | 12.0  (6.7-17.3)  150 | 19.2  (3.0-35.5)  26 |
| BC | 8.8  (7.6-10.0)  2,186 | 11.4  (10.3-12.4)  3,714 | 19.0  (17.2-20.9)  1,726 |
| BI | 12.3  (8.8-15.9)  333 | 19.8  (16.5-23.0)  587 | 22.1  (14.9-29.3)  131 |
| GE | 4.7  (1.3-8.1)  149 | 14.1  (10.5-17.6)  363 | 14.6  (7.6-21.5)  103 |
| OK | 8.9  (7.1-10.7)  947 | 14.3  (7.0-21.6)  91 | 80.0  NA  5 |
| ST | 9.5  (5.7-13.3)  232 | 18.0  (15.0-21.0)  622 | 20.0  (15.0-25.0)  250 |
| TU | 12.1  (7.5-16.6)  199 | 17.0  (12.8-21.2)  306 | 19.5  (12.6-26.5)  128 |
| VI | 14.3  (4.1-24.4)  49 | 23.2  (17.0-29.4)  181 | 25.0  (14.1-35.9)  64 |
| ALL | 9.3  (8.4-10.2)  4,273 | 13.7  (12.9-14.6  6,144 | 19.4  (17.9-21.0)  2,460 |
